# A-site cation engineering and halide tuning *via* precursor engineering to tune the optical properties of 2D perovskites

**DOI:** 10.1039/d5ra03422a

**Published:** 2025-08-08

**Authors:** Susana Ramos-Terrón, Cristina Martín, Gustavo de Miguel, Eduardo Solano, Daniel Hermida-Merino, Joris Van de Vondel, Johan Hofkens, Masoumeh Keshavarz

**Affiliations:** a Departamento de Química Física y Termodinámica Aplicada, Instituto Químico para la Energía y el Medioambiente (IQUEMA), Universidad de Córdoba, Campus de Rabanales Edificio Marie Cure E-14071 Córdoba Spain qf2rates@uco.es; b Department of Physical Chemistry, Faculty of Pharmacy, University of Castilla-La Mancha 02071 Albacete Spain; c NCD-SWEET Beamline, ALBA Synchrotron Light Source Cerdanyola del Vallès 08290 Spain; d CINBIO, Departamento de Física Aplicada, Universidade de Vigo, Campus Lagoas-Marcosende Vigo 36310 Spain; e Quantum Solid-State Physics (QSP), Department of Physics and Astronomy, KU Leuven 3001 Leuven Belgium; f Department of Chemistry, KU Leuven Celestijnenlaan 200F 3001 Leuven Belgium; g Leuven Institute for Micro- and Nanoscale Integration, Micro and Nano Systems 3001 Leuven Belgium masoumeh.keshavarz@kuleuven.be

## Abstract

The optical properties of the metal halide perovskites (MHPs) have been modulated by replacing the typical A-site cations or, alternatively, by using different halides (I, Br or Cl) in the chemical composition. In this study, a combined strategy involving A-site cation engineering and halide tuning *via* precursor engineering has been employed to investigate its impact on the structural and optical properties of BA_2_(MA_1−*x*_)_2_Pb_3_X_10_ (BA = butylammonium, MA = methylammonium, A = A-site cation, X = I, Br) two-dimensional (2D) perovskite. The substitution of guanidinium (Gua) and ethylammonium (EA) for methylammonium (MA), along with the use of Br instead of I as anions, was systematically analyzed. The obtained results demonstrate that these modifications significantly alter the dimensionality of the perovskite, favoring the formation of lower *n*-phases and resulting in blue-shifted absorption and photoluminescence due to quantum confinement. By adjusting the Gua/EA and I/Br content in the precursor solution, precise control of photoluminescence band position was achieved. The relevance of these findings was demonstrated as a proof-of-concept by fabricating light-emitting devices (LEDs), where the electroluminescence closely followed the compositional adjustments. Notably, Gua-based perovskites with Br anions exhibited enhanced LED performance due to optimal phase distribution. This research advances the understanding of structure–properties relationships and highlights the potential of composition engineering for innovative optical applications.

## Abstract 2

The optical properties of the metal halide perovskites (MHPs) have been modulated by replacing the typical A-site cations or, alternatively, by using different halides (I, Br or Cl) in the chemical composition.

## Graphical abstract

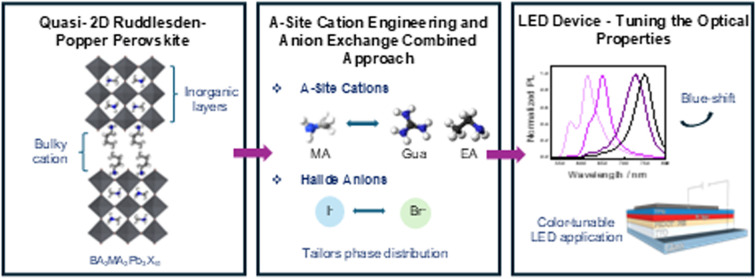

## Introduction

Metal halide perovskites (MHPs) have been extensively studied in recent years in the field of solar cells and light-emitting devices due to their unique electronic and optical properties.^1–7^ However, defect migration, environmental susceptibility, and halide segregation still remain as important weaknesses of these materials, in addition to the intrinsic low stability of the three-dimensional (3D) MHPs.^8–11^ To address these challenges, quasi-two-dimensional (2D) MHPs have emerged as promising alternatives due to their improved environmental stability. The chemical structure of the quasi-2D MHPs consists of featuring corner-sharing lead halide octahedra separated by organic spacer cations which can be tailored to achieve desired crystal structures and properties.^12–16^

Ruddlesden–Popper (RP) MHPs are a type of quasi-2D material that are derived from slicing the 3D perovskite structure along the (100) crystallographic plane. The chemical formula is A′_2_A_*n*−1_B_*n*_X_3*n*+1_,^13,17^ which consists of layers of corner-sharing metal halide octahedra [BX_6_]^4^, where B represents Pb^2+^ or Sn^2+^ and X is a halide anion (I/Cl/Br). The perovskite framework contains A-sites, which are occupied by monovalent cations such as methylammonium (MA), formamidinium (FA), or cesium (Cs).^18–20^ In the case of the *n* term, it represents the nominal stoichiometry, indicating the number of packed inorganic layers, which determines the dimensionality (thickness) and, consequently, changes the band gap energies.^21–24^ Thus, the optoelectronic properties are closely related to the chemical structure, where the number of inorganic layers stands out as a significant factor, directly affecting the quantum confinement of the photogenerated charge carriers. Controlling the ratio of the monovalent anion (I/Cl/Br) is the most popular way to modify the band gap,^25–27^ but this approach is very challenging due to the mixed halide composition, which often leads to phase separation and ion migration, compromising the operational stability of the devices.^28–30^ To overcome these limitations, it has recently been demonstrated that the A-site cations not only allow the tuning of the optoelectronic properties by modifying the distribution of the *n*-phases but also help to suppress the halide segregation.^31–33^

Based on this concept, the role of different cations (guanidinium, Gua, and ethylammonium, EA) in combination with methylammonium (MA) as the A-site cation in BA_2_(MA_1−*x*_A_*x*_)_2_Pb_3_I_10_ 2D RP perovskites has been previously investigated.^34^ The structural analysis together with the optoelectronic properties of these materials have demonstrated the ability of the A-cation approach to tailor *n*-phases in 2D RP MHPs.

Although halide composition adjustment and A-site cation tuning have each been studied to enhance perovskite properties, their combination within a controlled synthesis strategy remains largely unexplored.^35^ In our previous work, we examined A-site cation substitution in iodide-based RP perovskites, focusing on the resulting structural and photophysical changes. However, a systematic study integrating both variables—cation and halide composition—in 2D RP systems has not yet been reported. Here, we address this gap by investigating how the simultaneous tuning of A-site cations (MA, Gua, EA) and halides (I, Br) affects the structural dimensionality, phase distribution, and optical response of BA_2_(MA_1−*x*_A_*x*_)_2_Pb_3_X_10_ films. This dual approach provides an effective route to tailor photoluminescence and electroluminescence across the visible spectrum, with direct implications for the development of tunable perovskite-based LEDs.

**Scheme 1:**
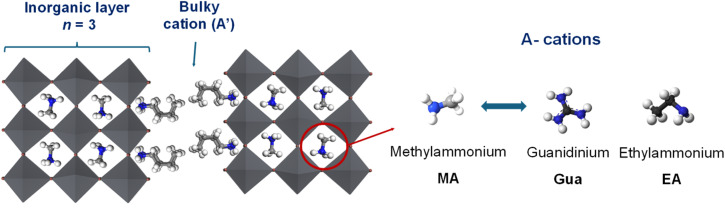
Schematic representation of the Ruddlesden–Popper perovskite structure with *n* = 3, illustrating the layered arrangement of PbX_6_ octahedra, the insertion of bulky A′-site cations between perovskite slabs, and the substitution of methylammonium (MA) at the A-site by guanidinium (Gua) or ethylammonium (EA).

Therein, we have combined A-site cation engineering with halide tuning *via* precursor engineering to finely tune the optical properties of the 2D RP BA_2_(MA_1−*x*_A_*x*_)_2_Pb_3_X_10_ (BA = butylammonium, MA = methylammonium) MHP. Thus, the MA cation was partially replaced in the precursor solution by the guanidinium (Gua) and ethylammonium (EA) cations in both I- and Br-based perovskites. The changes in the absorption and photoluminescence spectra were studied upon 30%, 60% and 90% substitution of the MA cation. The shift to lower wavelengths was ascribed to a change in the phase distribution due to the larger size of the Gua and EA cations in both I- and Br-based perovskites. Light-emitting devices were fabricated as a proof-of-concept to demonstrate the ease of preparation of precise colour perovskite-based light-emitting devices (Scheme 1).

## Results and discussion

### Structural characterisation and orientation

**Fig. 1:**
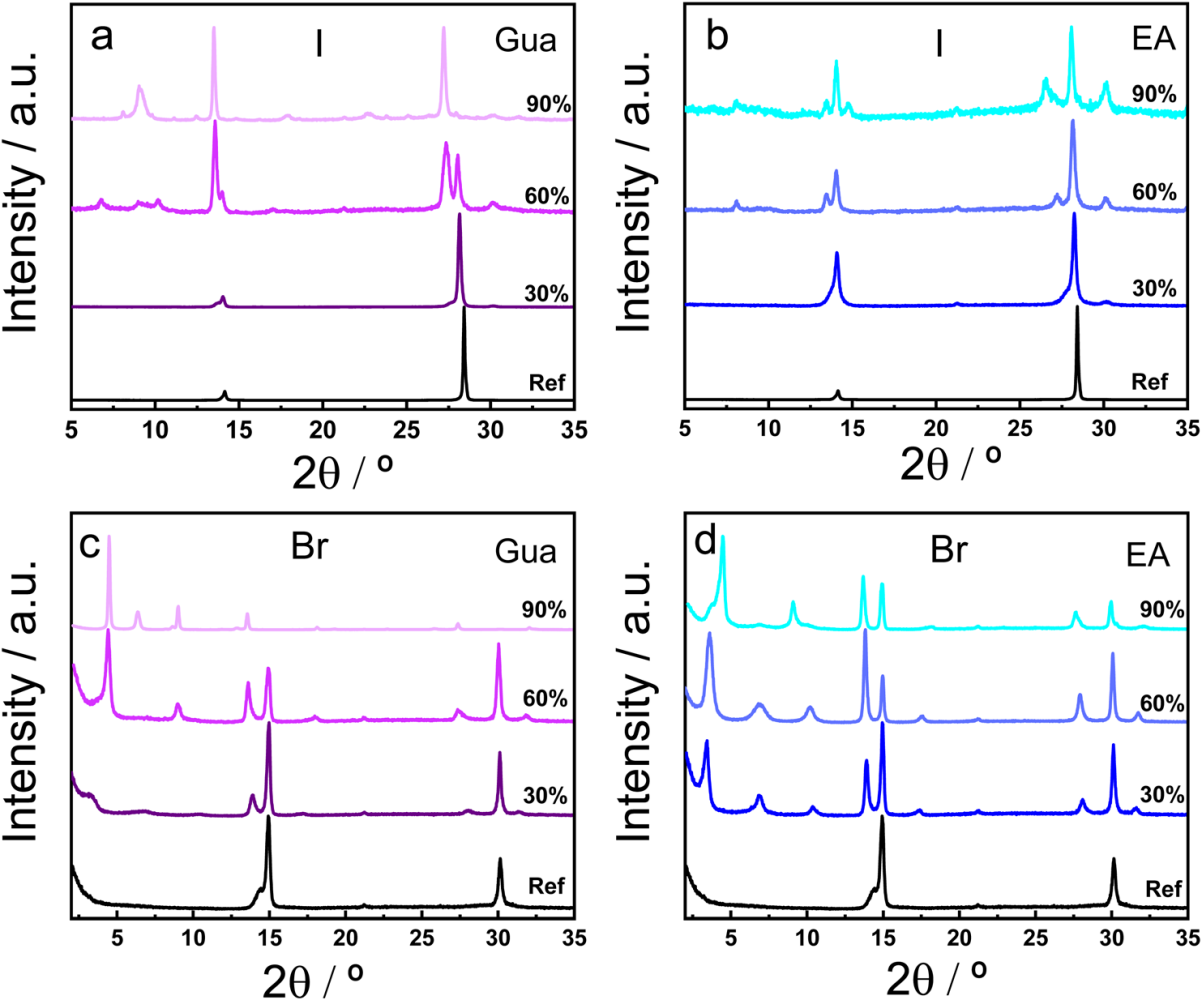
Normalized X-ray diffraction (XRD) patterns recorded at Cu K_α_ wavelength of the BA_2_(MA_1−*x*_A_*x*_)_2_Pb_3_X_10_ 2D RP MHP thin films, being X = I in (a and b) and Br in (c and d). Different percentages of the Gua cation (a and c) and EA cation (b and d) were also recorded.

The iodide- and bromide-based two-dimensional (2D) Ruddlesden–Popper (RP) metal halide perovskites (MHPs) thin films were synthesized by using the spin-coating method.^34^ The structural characterization of the BA_2_(MA_1−*x*_A_*x*_)_2_Pb_3_X_10_ MHP (BA = *n*-butylammonium, MA = methylammonium, A = guanidinium, Gua, or ethylammonium, EA, and X = I or Br) was carried out using X-ray diffraction (XRD) and synchrotron grazing-incidence wide-angle X-ray scattering (GIWAXS) measurements. Fig. 1 displays the XRD patterns of the I- and Br-based 2D RP MHPs films with different percentages of the Gua and EA cations. The XRD patterns of the films with only MA as the A-cation display only two Bragg peaks at 14.04°/28.32° and 14.94°/30.14° for the I- and Br-based materials, respectively. In both 2D RP MHPS, the reflection peaks correspond to the (111) and (202) crystallographic planes. The shift of the Bragg peaks to larger 2*θ* values in the Br-based perovskites reflects a decrease in the interplanar spacing of the corresponding crystallographic planes, consistent with the smaller ionic radius of Br^−^ compared to I^−^. Moreover, the variation of the relative intensity of the Bragg signals in the I- and Br-based materials reveals an unlike orientation of the MHPs layers.

The XRD patterns of the films with increasing percentage of the Gua and EA cations (from 30% to 90%) display two major changes with respect to the reference films: a shift in the position of the (111) and (202) peaks to lower 2*θ* values for both halide perovskites (Fig. S1) and the appearance of new reflection peaks, specially at low 2*θ* angles, which is more evident at high A-cation replacement and, in particular, in the Br-based 2D RP perovskite. The Bragg peak shift towards lower 2*θ* values signals a partial incorporation of the Gua and EA cations into the octahedral voids of the 3D domains where the larger size of the former A-cations cause an expansion of the unit cell.^36,37^ The new XRD peaks at 9.12° (002)/13.60° (003)/27.33° (006) and 6.91° (002) are assigned to the formation of the *n* = 2 and *n* = 3 phases in the I-based 2D RP MHP, respectively, while the peaks at 6.9° (002) and 4.5° (001)/9.05° (003)/13.56° (004) are attributed to the *n* = 1 and *n* = 2 phases in the Br-based materials, respectively.^34^ The identification of *n* = 1, 2, and 3 phases was supported by characteristic reflections observed in both XRD and GIWAXS patterns, consistent with previously reported assignments for RP structures with similar composition.^34,39^ Thus, at low percentage of the Gua and EA cations (30%), these are initially incorporated into the octahedra voids replacing the MA cations in all *n*-phases but, at high content of Gua and EA, the low dimensional phases (*n* = 1, 2 and 3) are preferentially formed. Notably, no evidence of a 1D GuaPbI_3_ phase is observed, indicating that the RP framework stabilizes layered structures even at high Gua concentrations. This result further confirms that Gua and EA cations preferentially incorporate into the A-site of the RP framework. Although their potential use as A′-site cations could be envisioned, previous reports and our own observations suggest that such substitution leads to destabilization of the layered structure, making them unsuitable as BA replacements. For this reason, this possibility was not pursued in the present study. In the Br-based perovskites, the smaller size of the unit cell diminishes the percentage of the Gua and EA that can be incorporated into the voids and, therefore, the low dimensional phases are formed at lower percentages of the A-cation. Thus, the replacement of the MA cation by the Gua or EA cations in the precursor solution allows one to control the dimensionality of the phases that are formed in the films.

**Fig. 2:**
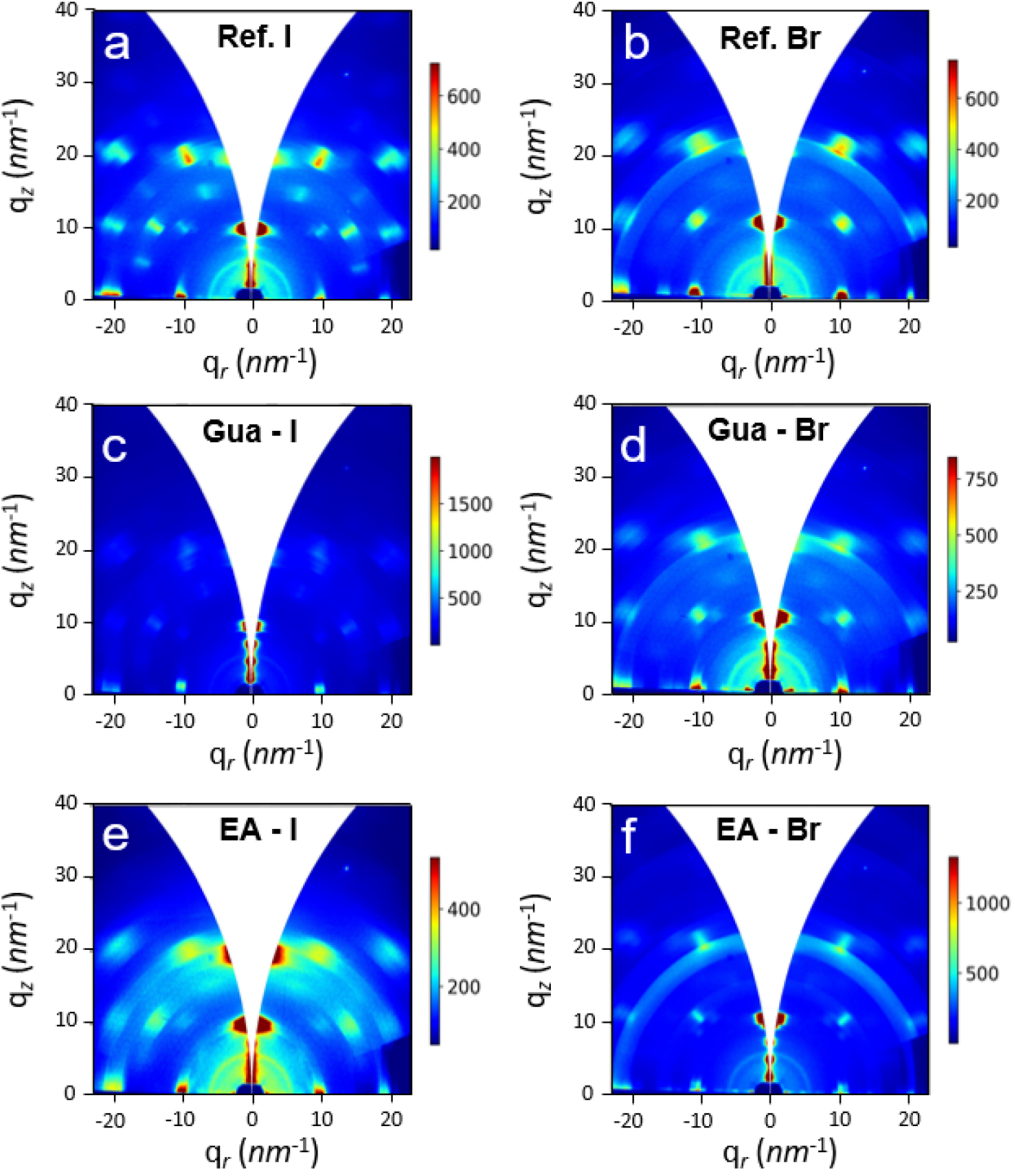
2D GIWAXS patterns of BA_2_MA_2_Pb_3_I_10_ (a) and BA_2_MA_2_Pb_3_Br_10_ (b) thin films. Panels (c and d) show the 2D GIWAXS patterns of BA_2_(MA_0.40_Gua_0.60_)_2_Pb_3_I_10_ and BA_2_(MA_0.40_Gua_0.60_)_2_Pb_3_Br_10_, respectively. Panels (e and f) correspond to the 2D GIWAXS patterns of BA_2_(MA_0.40_EA_0.60_)_2_Pb_3_I_10_ and BA_2_(MA_0.40_EA_0.60_)_2_Pb_3_Br_10_, respectively.

Synchrotron GIWAXS measurements were employed to evaluate changes in the orientation of the 2D RP MHPs upon addition of the Gua and EA cations. GIWAXS data provides structural and orientation information of the investigated 2D materials. Fig. 2 shows the calibrated 2D images for the I- and Br-based BA_2_MA_2_Pb_3_X_10_ 2D RP HMPs as well as those with 60% replacement of the MA cation by the EA or Gua cations. The 2D images of the reference perovskites display Bragg spots instead of diffraction rings which indicates a preferential orientation of the film, as expected for a quasi-2D system.^38^ The intense out-of-plane (*q*_r_ = 0 nm^−1^) Bragg spot at *q*_z_ = 10 nm^−1^ in both films is attributed to the (111) reflection of the *n* = ∞ phase. Upon A-site substitution, additional diffraction spots are observed at *q*_z_ ≈ 6.3 nm^−1^, 4.6 nm^−1^, and 3.2 nm^−1^, which correspond to the (002), (003), and (004) reflections of lower-*n* RP phases (*n* = 2 and 3), respectively. These spots, also located at *q*_r_ = 0 nm^−1^, confirm the formation of low-dimensional perovskite phases with preserved out-of-plane orientation.

The orientation of the Bragg spots in the 2D images account for a stacking of the octahedra layers assembled in-plane, with an out-of-plane orientation, *i.e.* parallel to the substrate.^38^ The incorporation of 60% of Gua or EA to the films induces subtle changes in the intensity of the Bragg spots. Thus, the addition of 60% Gua to the I-based perovskite or 60% EA to the Br-based perovskite decreases the intensity of the Bragg spots out of *q*_r_ = 0, but it significantly rises the intensity of the peaks at low *q*_z_ and *q*_r_ = 0 which, in both cases, is associated to the formation of lower dimensional perovskites. In summary, the minor changes in the GIWAXS signals are ascribed to the modification of the distribution of the *n*-phases but not to changes in the orientation of the films (as proved by the constant Bragg peaks position) which, in all cases, maintain the octahedra layers parallel to the substrate. While both techniques indicate a preferred (111) orientation, GIWAXS provides complementary structural information beyond conventional Bragg–Brentano XRD. In particular, GIWAXS confirms the preservation of out-of-plane orientation and reveals subtle changes in the distribution of low-*n* phases (*n* = 2 and 3), based on differences in intensity and the presence of low-*q* reflections. Although these phases are also observed in the XRD patterns, their identification is limited by overlapping peaks and texture effects. GIWAXS, with its angular resolution and 2D detection, strengthens the phase assignment and orientation analysis. While the formation of low-*n* phases is also observed by XRD, GIWAXS measurements confirm their presence and relative distribution with greater clarity, due to its higher angular resolution and sensitivity to out-of-plane orientation.

### Optical properties

**Fig. 3:**
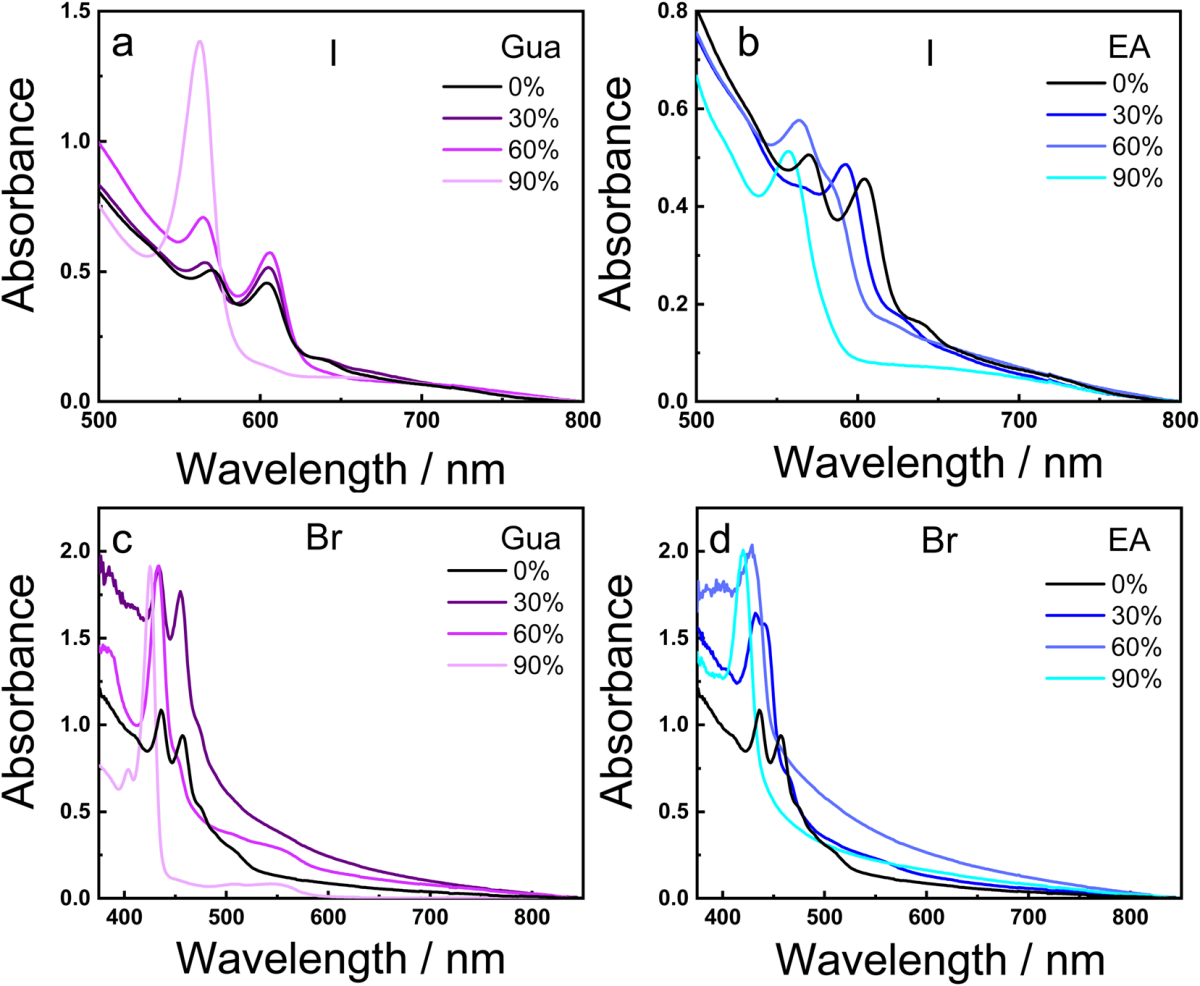
Absorption spectra of the I-based (a and b) and Br-based (c and d) BA_2_(MA_1−*x*_A_*x*_)_2_Pb_3_X_10_ MHP thin films at different percentages of the Gua (left panels) and EA (right panels) cations.

The optical properties of the mixed A-cation 2D RP MHPs were investigated by using UV-vis absorption and photoluminescence (PL) spectroscopy. Fig. 3 displays the absorption spectra for all mixed A-cation 2D RP BA_2_(MA_*x*_A_1−*x*_)_2_Pb_3_X_10_ MHPs. In the I-based materials, the films with only the MA cation displays absorption peaks around 570 nm and 610 nm which corresponds to the *n* = 2 and *n* = 3 phases, respectively.^34^ In the Br-based perovskites, sharp absorption peaks around 436 and 457 nm are attributed to the *n* = 2 and *n* = 3 phases, respectively.^39^ The weak absorption tail in both I- and Br-based reference sample (100% MA) at long wavelengths is associated to phases with high *n* values (*n* = ∞). The increasing amount of the Gua and the EA cations in the I-based materials reveals an enhancement of the absorption signals related to the *n* = 2 and *n* = 3 phases, ending up in films with 90% of Gua or EA where only the *n* = 2 phase is visible. The sharp absorption bands observed in low-*n* phases are also known to involve strong excitonic contributions, as reported in previous studies.^39,42^ However, in the Br-based perovskites, the addition of moderate amount of Gua (30% and 60%) entails the appearance of absorption in the 475–575 nm region associated with intermediates *n*-phases. On the contrary, the addition of EA only involves the formation of lower *n*-phases (*n* = 1 and 2).

**Fig. 4:**
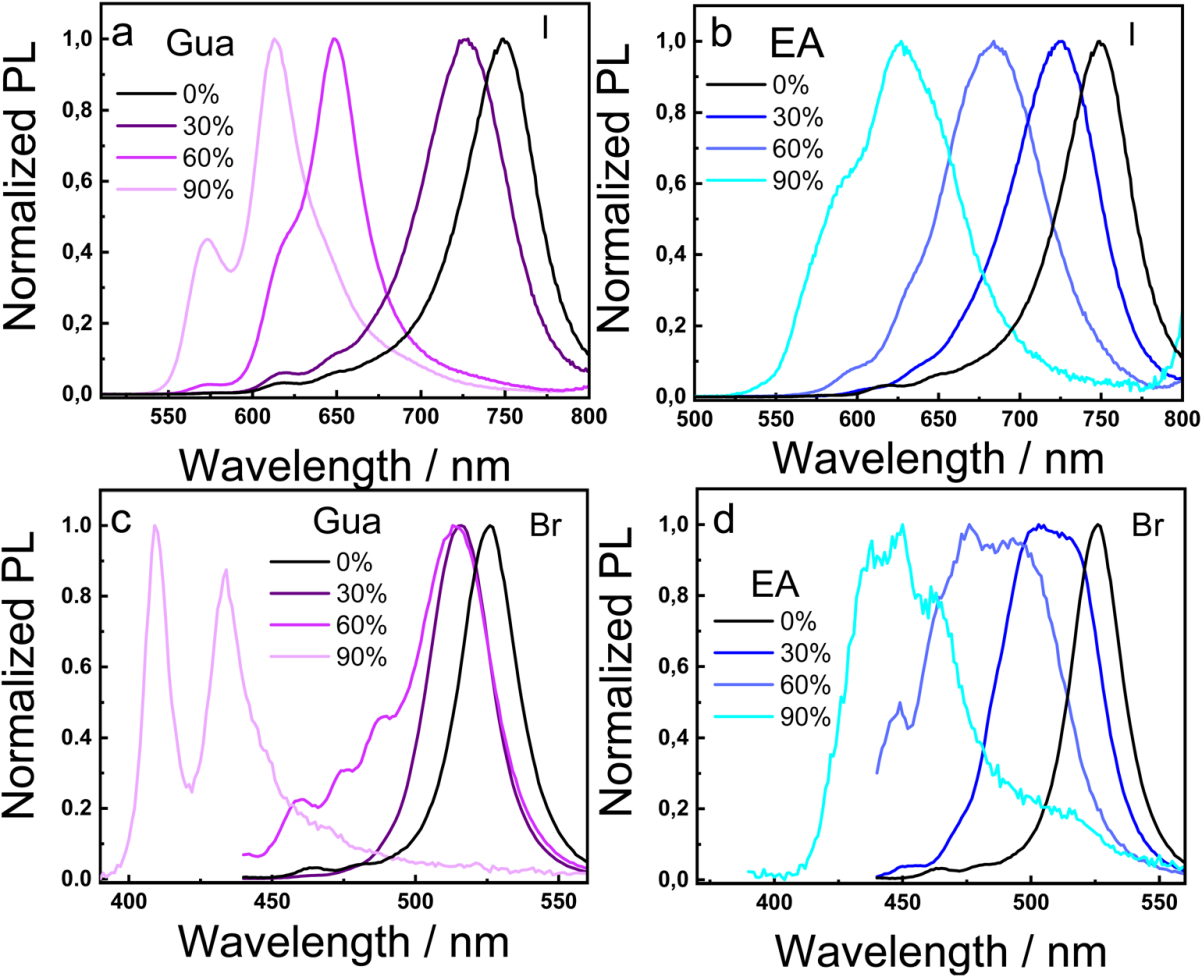
Normalized photoluminescence (PL) spectra of BA_2_(MA_1−*x*_A_*x*_)_2_Pb_3_X_10_ thin films with increasing A-site cation substitution. Panels (a and b) correspond to I-based perovskites containing Gua and EA, respectively, while panels (c and d) show the Br-based analogues. A progressive blue-shift in the emission peaks is observed as the percentage of Gua or EA increases, consistent with the formation of lower-*n* phases.

Fig. 4 shows the normalized emission spectra for the 2D RP MHP films. The PL spectra of the reference film with only MA displays an emission band centered at 750 nm, characteristic of the *n* = ∞ phase, due to the energy funneling mechanism.^40^ In the case of the I-based perovskites, Fig. 4a shows that increasing the Gua content causes a progressive blue shift in the PL emission, shifting from 750 nm to 670 nm, 650 nm and 610 nm as the Gua concentration increases, due to the formation of lower-*n* phases (*n* = 4, 3 and 2, respectively). A similar trend is observed in Fig. 4b for the I-based films with EA. This blue shift in the PL matches perfectly with the formation of the low-dimensional phases detected in the XRD and absorption measurements. Regarding the Br-based perovskites, the PL emission centred at 525 nm at 100% MA is attributed to the phase with *n* = ∞.^39^ As shown in Fig. 4c, the addition of Gua leads to the appearance of higher-energy emission. The shift is moderate at 30% and 60% Gua, but becomes more pronounced at 90%, where the signal at 409 nm is associated with the *n* = 1 phase. Thus, the Gua cation seems to accommodate well in the octahedral voids at 30% and 60% substitution, without disturbing the 2D structure and the nature of the low-*n* phases. However, at 90%, its larger size only allows the formation of the *n* = 1 and 2 phases. In the case of the Br-based perovskites with the EA cation (Fig. 4d), the PL shift is more gradual, which accounts for a smooth incorporation of the EA replacing the MA, without largely disturbing the 2D structure.

Table S1 depicts the photoluminescence quantum yield (PLQY) at a very low excitation intensity of 1 mW cm^−2^.^41,42^ The PLQY reaches a maximum value of 5% for the Br-based perovskite, which is explained due to an enhancement of exciton formation due to quantum confinement facilitating the radiative recombination.^39,42^ The addition of 60% Gua or EA to the Br-based perovskite largely modifies the PLQY, reducing the value to 0.5% with EA and increasing the PLQY to 17% with Gua. The changes in the PLQY upon addition of the EA and Gua cation are attributed to the changes in the *n*-phases. Thus, the increase of the PLQY with Gua is related to an increase of the concentration of the *n* = 4 phase, with higher QY.^39^ The decrease of the PLQY with EA is associated to the lower QY of the phases with low *n* values. In addition, nonradiative recombination processes may also contribute to this behavior. EA-containing compositions may suffer from enhanced trap-assisted recombination or increased defect densities, which are more likely in low-*n* phases and could further reduce their radiative efficiency. Regarding the I-based perovskites, the almost no change in the PLQY upon addition of the 60% Gua or EA cations is related to the small modification of the *n* values at this percentage of the A-cations.

The kinetics of the excited state of the I- and Br-based perovskites were studied by using picosecond time-resolved PL spectroscopy upon excitation at 405 nm with a fluence of 1.5 nJ cm^−2^ (see Fig. S2). In the I-based materials, the addition of Gua or EA (60%) does not change the PL decays in good agreement with the similar PLQY. In the Br-based perovskites, a much longer PL decay for the sample with 60% of Gua is observed, matching well with the larger PLQY and being attributed to a different distribution of *n*-phases and consequently to a variation in the exciton binding energy.^34^

### Perovskite light-emitting diodes (PeLEDs)

**Fig. 5:**
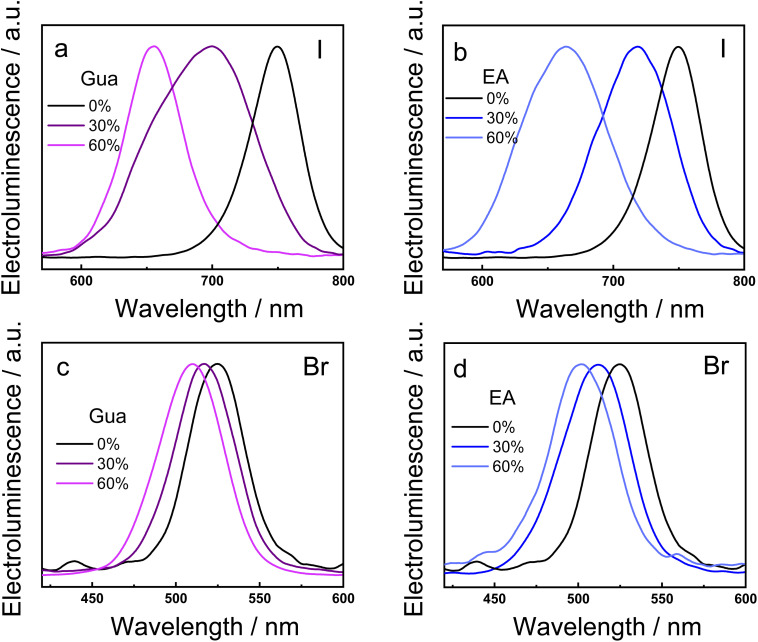
Electroluminescence spectra of light-emitting diodes fabricated with BA_2_(MA_1−*x*_A_*x*_)_2_Pb_3_X_10_ 2D RP MHP thin films. The halogen X atoms are I in (a and b) and Br in (c and d). The A-cations are Gua in (a and c) and EA in (b and d).

The mixed A-cation and anion strategy has demonstrated to be successful in finely shifting the position of the PL peak. To exploit this effect in optoelectronic applications, simple light-emitting diodes were fabricated using as the active layers the I- and Br-based 2D RP MHPs with different percentage of the Gua and EA cations. Fig. 5 displays the electroluminescence spectra for all PeLEDs, covering a broad range of the visible region, from 475 to 800 nm depending on the percentage of the A-cation substitution and the type of halide atom. In the I-based perovskites, the replacement of 30–60% of MA by Gua or EA allows to control the electroluminescence in the 600–800 nm region. The electroluminescence peaks are generally broad due to emission of phases with different *n* value. For example, the film with 30% of Gua displays signal from 600 nm to 750 nm due to emission from *n* = 2, *n* = 3, *n* = 4 and *n* = 5 phases. In the Br-based perovskites, the A-cation substitution leads to electroluminescence signals in the 475–550 nm region, depending on the percentage of the Gua or EA cation. It is worth noting that the position of all electroluminescence spectra is in agreement with the PL bands, strongly indicating that the electron–hole recombination mechanism remains the same under light or voltage excitation.

**Fig. 6:**
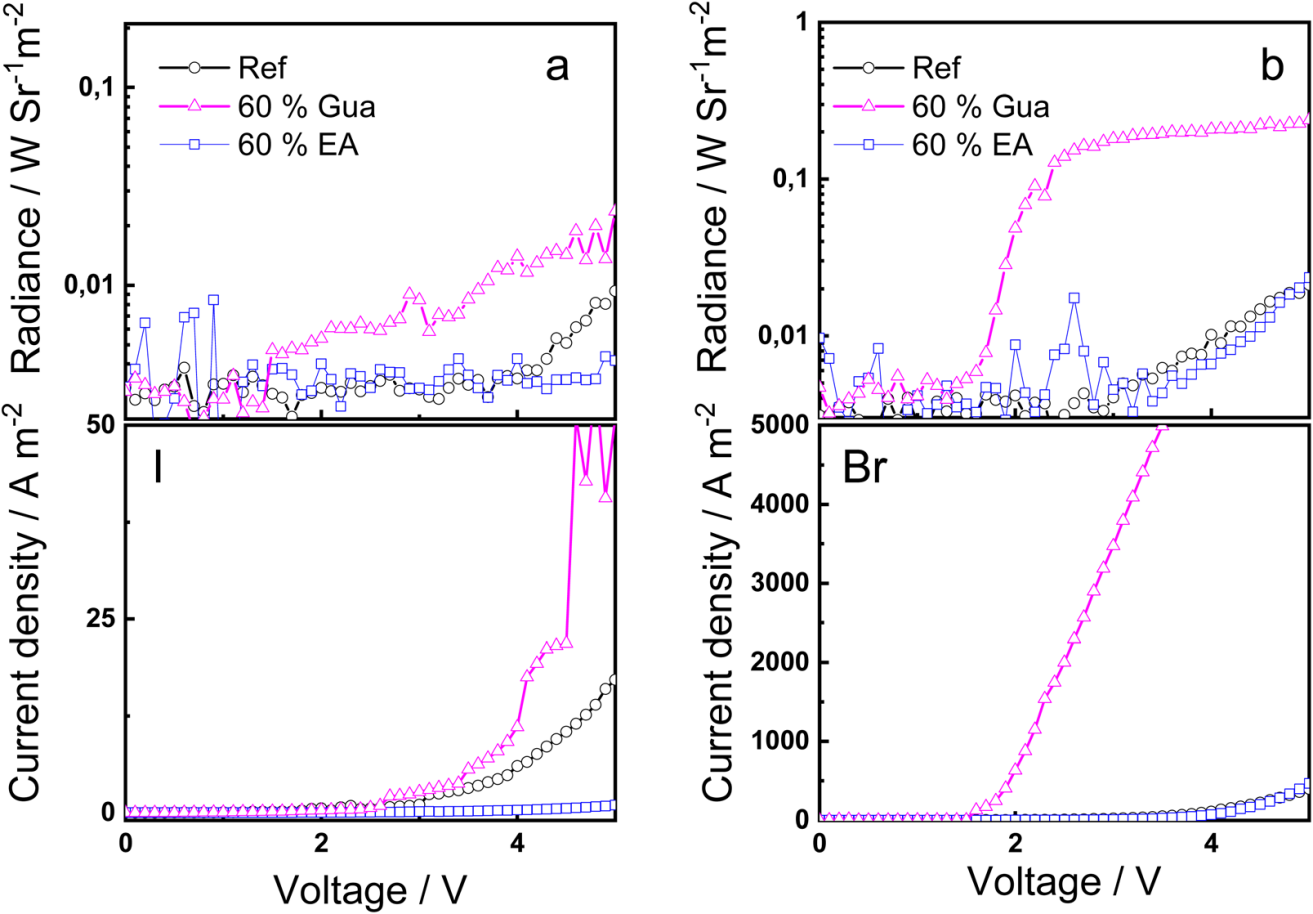
*J*–*V* curve and luminance of (a) BA_2_(MA_0.40_A_0.60_)_2_Pb_3_I_10_ and (b) BA_2_(MA_0.40_A_0.60_)_2_Pb_3_Br_10_ MPH-based devices.

Fig. 6 displays the radiance and current density of the PeLEDs (only 60% of the A-cation exchange) where the best radiance and lower turn-on voltage are obtained for the device containing the Gua cation and the Br anion. The replacement of the MA by the EA cation did not modify the performance of the devices respect to the reference 2D RP MHP. Interestingly, the Gua substitution has a greater impact on the PeLED performance than the Br replacement. The role of the cation exchange is explained on a modification of the distribution of *n*-phases (quantum confinement) which increases the PLQY. Although absolute values of external quantum efficiency (EQE) and luminance in cd m^−2^ could not be determined, the radiance values enable a meaningful comparison of relative device performance across compositions.

## Conclusions

In summary, this study employed several experimental techniques to thoroughly investigate the influence of A-site cations engineering combined with halide tuning *via* precursor engineering on the on the structural and optical properties of the BA_2_(MA_1−*x*_A_*x*_)_2_Pb_3_X_10_ 2D RP MHP.

The incorporation of the Gua and EA cations in both the I- and Br-based perovskites modifies the dimensionality of the material, favoring the formation of phases with low *n* values while keeping its preferential out-of-plane orientation. Thus, the absorption and photoluminescence signals are blue-shifted due to the quantum confinement effect in low dimensional perovskites. Interestingly, a precise control of the PL band position can be achieved by adjusting the ratio of Gua or EA and the I/Br content in the initial precursor solution.

The pertinence of these results has been evaluated as a proof-of-concept by fabricating light-emitting devices (LEDs). Thus, similarly to the PL signals, the PeLEDs exhibit electroluminescence signals according to the Gua and EA and I/Br content. In particular, in the Br-based perovskites, the devices containing Gua displays improved LED performance as a result of optimal phase distribution with intermediates *n* phases.

This work goes beyond previous studies by combining two complementary compositional strategies—A-site cation engineering and halide tuning—within a unified precursor formulation. While each approach has been individually explored in 2D perovskites, their combined effect on phase control, quantum confinement, and optical tunability had not been addressed until now. Our results demonstrate that this dual strategy enables fine control over the distribution of low-*n* phases, leading to a reproducible tuning of photoluminescence and electroluminescence across the visible range. This provides a versatile and scalable method for designing color-selectable perovskite-based optoelectronic devices. This research represents a significant step forward in the structure/property relationship of the MHPs allowing novel optical applications by precise composition engineering.

## Author contributions

Susana Ramos-Terrón: methodology, investigation, writing – original draft, visualization. Cristina Martín: conceptualization, methodology, investigation, writing – original draft. Gustavo de Miguel: resources, writing – review & editing, funding acquisition. Eduardo Solano: validation, formal analysis. Daniel Hermida-Merino: validation, formal analysis. Joris Van de Vondel: investigation, formal analysis. Johan Hofkens: resources, funding acquisition. Masoumeh Keshavarz: conceptualization, methodology, investigation, writing – original draft.

## Conflicts of interest

There are no conflicts to declare.

## Supplementary Material

RA-015-D5RA03422A-s001

## Data Availability

The data for this article, including: X-ray powder diffraction (XRD), synchrotron grazing incidence wide-angle X-ray scattering (GIWAXS) measurements, ultraviolet-visible (UV-vis) absorption and photoluminescence (PL) spectroscopy, electroluminescence spectra, current–voltage (*J*–*V*) curve and luminance are available at https://helvia.uco.es/handle/10396/29649 and are provided in user-accessible formats. The supplementary information file includes details on the materials and methods used for the synthesis and deposition of 2D perovskite films and PeLED devices. It also contains additional characterization data such as XRD, GIWAXS, UV-vis absorption, steady-state and time-resolved photoluminescence, quantum yield, and electrical measurements, as well as supporting figures and tables. See DOI: https://doi.org/10.1039/d5ra03422a.
