# Supplementary material for: A-site cation engineering and halide tuning via precursor engineering to tune the optical properties of 2D perovskites
Source: RSC Adv. 2025 Aug 8;15(34):28181–90. doi: 10.1039/d5ra03422a (PMC12377037; doi:10.1039/d5ra03422a)
Supplement: RA-015-D5RA03422A-s001 [file RA-015-D5RA03422A-s001.pdf]

Supplementary Information (SI) for RSC Advances.
This journal is © The Royal Society of Chemistry 2025

Supporting information

A-Site Cation Engineering and Halide Tuning via Precursor Engineering to Tune the Optical Properties of 2D Perovskites

Susana Ramos-Terrón,[*,a] Cristina Martín,[b] Gustavo de Miguel,[a] Eduardo Solano,[c] Daniel Hermida-Merino,[d] Joris Van de Vondel,[e] Johan Hofkens,[f] Masoumeh Keshavarz [*,g]

[a]. Departamento de Química Física y Termodinámica Aplicada, Instituto Químico para la Energía y el Medioambiente (IQUEMA), Universidad de Córdoba, Campus de Rabanales, Edificio Marie Cure, E-14071 Córdoba, Spain

[b]. Department of Physical Chemistry, Faculty of Pharmacy, University of Castilla-La Mancha, 02071, Albacete, Spain.

[c]. NCD-SWEET Beamline, ALBA Synchrotron Light Source, Cerdanyola del Vallès, 08290, Spain.

[d]. CINBIO, Departamento de Física Aplicada, Universidade de Vigo, Campus Lagoas-Marcosende, Vigo 36310, Spain.

[e]. Leuven Institute for Micro- and Nanoscale Integration, 3001 Leuven, Belgium; Micro and Nano Systems, 3001, Leuven, Belgium.

[f]. Department of Chemistry, KU Leuven, Celestijnenlaan 200F, 3001 Leuven, Belgium.

[g]. Quantum Solid-State Physics (QSP), Department of Physics and Astronomy, KU Leuven,3001, Leuven, Belgium.

*Corresponding authors, email address: qf2rates@uco.es, masoumeh.keshavarz@kuleuven.be

Starting Materials:

Lead iodide (PbI_2, 99%), lead bromide ($PbBr_2$, 99%), methylammonium iodide (MAI, 98%), methylammonium chloride (MACl, 98%), *n*-butylammonium iodide (BAI, 99%), [6,6]-Phenyl C_{61} butyric acid methyl ester (PCBM), poly(9-vinylcarbazole) (PVK), 2,2',2''-(1,3,5-Benzinetriyl)-tris(1-phenyl-1-H-benzimidazole) (TPBi), Poly(3,4-ethylenedioxythiophene)-poly(styrenesulfonate) (PEDOT), and toluene (99.8%) were obtained from Sigma-Aldrich. *N,N*-dimethylformamide (DMF, 99.8%), extra dry over Molecular Sieve, AcroSeal, was sourced from Acros Organics.

Glass substrates patterned with fluorine tin oxide (FTO), indium tin oxide (ITO) and amorphous glass were provided by Pilkington. For the cleaning process, absolute dry ethanol (maximum 0.02% water) and isopropyl alcohol (technical grade, 99.5%) were purchased from PanReac, and Decon 90 soap was acquired from Decon.

Substrate Preparation:

All substrates underwent a sequential cleaning process involving sonication in Decon 90, Milli-Q water, ethanol, and isopropyl alcohol, followed by a 15-minute ultraviolet-ozone treatment.

Perovskite Solution Preparation:

Stoichiometric precursor solutions were prepared by mixing PbI_2/$PbBr_2$, MAI/MACl, Gua/EA and BAI, in 0.5 mL of DMF following the steps detailed in the previous works.[1,2]

Perovskite Deposition:

The perovskite films were deposited by the hot casting (HC) method.[1,2] The substrates were heated at 90 °C for 10 min. Then, they were moved quickly on the spin-coater and 50 µL of the precursor solution was deposited on the substrates at 5000 rpm for 30 s. Finally, the films were annealed at 100 °C for 20 min on a hot plate.

Perovskite light emitting devices (PeLEDs):

The structure device given ITO/PEDOT:PSS/PVK/perovskite/TBPi/Al was fabricated. A PEDOT:PSS layer (40 nm in thickness) was deposited onto the ITO glass substrate by spin-coating (5000 rpm for 50 s) using 200 µL of solution. Then, PEDOT:PSS layer was kept at 145 ºC for 15 min. Later, PVK layer (40 nm) was synthesized using a toluene solution (10 mg in 1 mL). 100 µL of PVK solution was deposited onto the PEDOT:PSS layer by spin-coating (3000 rpm, 60 s) and annealed at 140 °C for 30 min. The perovskite films were deposited by the HC method as indicated above. After this, TBPi was spin coated at 5000 rpm, for 60 s from a toluene solution (10 mg in 1 mL) and annealed for 10 min at 100 ºC. Finally, a 40 nm aluminium electrode was deposited as a metallic contact by thermal evaporation (1×10^{-6} torr.).

X-ray Diffraction (XRD) Measurements:

XRD experiments were conducted on all samples using a Bruker D8 DISCOVER diffractometer operating at 40 kV and 40 mA, with Cu Kα radiation (λ = 1.54060 Å). The measurements were taken within the range of 2 to 40° Bragg angles. A step size of 0.015° and a counting time of 1.5 s per step were used.

Synchrotron-based Grazing Incidence Wide Angle X-ray Scattering (GIWAXS):

GIWAXS data was collected at two different beamlines BM26 DUBBLE beamline (ESRF) and NCD-SWEET beamline (ALBA):

BM26 DUBBLE beamline: The bending magnet produced X-ray beam was monochromatized to 12 keV (1.033 Å) using a Si (111) double crystal monochromator and focused on the detector using a KB system. A FReLoN 2k CCD camera of 2048 x 2048 pixels with a pixel size of 48 x 49 μm^2 [horizontal × vertical] was employed as X-ray detector.

NCD-SWEET beamline: An X-ray beam produced by an *in vacuo* undulator was monochromatized using a Si (111) channel-cut monochromator at 12.4 keV (λ = 0.9998 Å). The X-ray beam was also collimated using an array of Be lenses, resulting in a beam size of 150 × 50 μm^2 [horizontal × vertical] at

the sample position. The two-dimensional images were recorded using a Rayonix® LX255-HS area detector.

The detector-to-sample distance, tilts, and reciprocal q-space were calibrated using Cr_2O_3 as standard. Data were analyzed using a custom Python routine.

UV–Vis Absorption Spectroscopy:

UV–Vis absorption spectra were measured at room temperature (25 °C) using a Cary 100 UV–Vis spectrophotometer within the wavelength range of in the range of 500–800 nm for all samples.

Steady-State and Time-Resolved Photoluminescence (PL) Measurements:

Steady-state photoluminescence spectra were recorded using an FLS980 (Edinburgh Instruments) photoluminescence spectrometer equipped with a 450 W Xenon arc lamp and an R298P photomultiplier as the detector. For time-resolved fluorescence measurements, the time-correlated single photon counting (TCSPC) technique was employed with the same FLS980 (Edinburgh Instruments) photoluminescence spectrometer. The thin films were excited at 406.4 nm using an 86.8 ps pulse width diode laser, and detection was performed with an R2658P photomultiplier. Temperature dependence PL measurements were carried out using a Optistat DN cryostat from Oxford instruments.

PL Quantum Yield Measurements:

The PLQYs were measured using an integrating sphere coupled to an FLS980 photoluminescence spectrometer. The FLS980 was equipped with double monochromators, a 450 W Xenon lamp and a PMT-R2658P detector. A neutral density filter (OD = 3) was placed in the emission path to measure the excitation region of the spectrum without detector saturation effects. The excitation was performed at 450 nm. The system was calibrated using a standard reference sample, and the estimated error of the PLQY values is ±0.3%.

Device Characterization:

Current–voltage curves were measured with a Keithley 2400 potentiostat. The PeLEDs and single carrier devices were masked with a metal aperture of 0.09 cm^2 to define the active area.

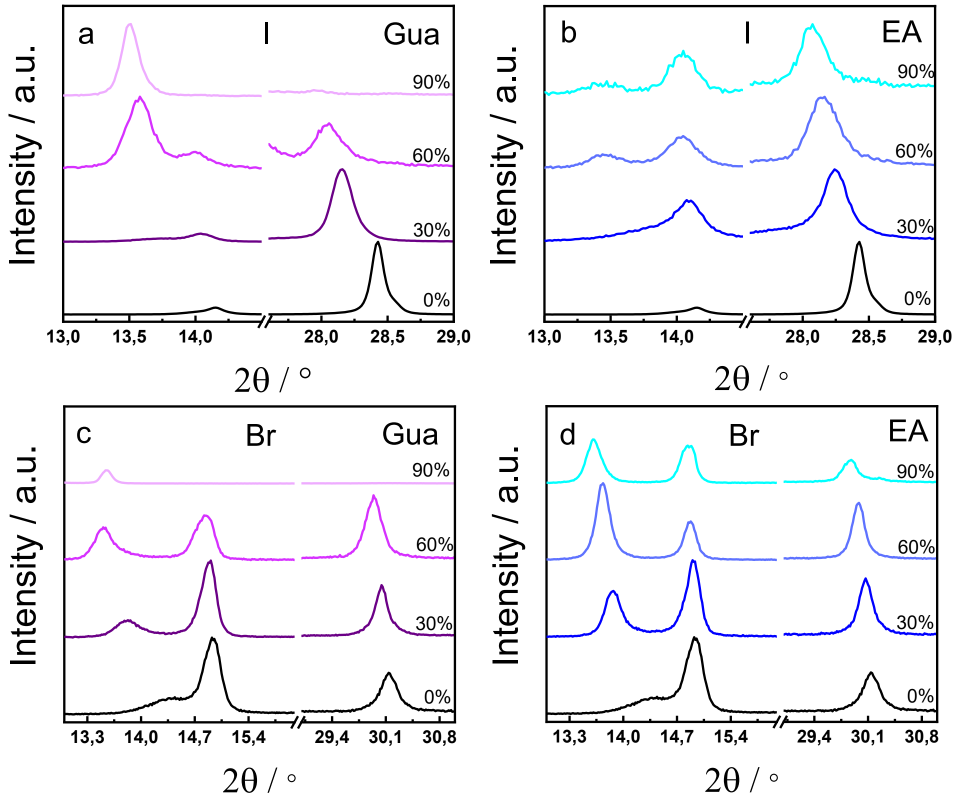

Figure S1. Magnified XRD patterns of a) $BA_2(MA_{1-x}Gua_x)_2Pb_3I_{10}$, b) $BA_2(MA_{1-x}EA_x)_2Pb_3I_{10}$, c) $BA_2(MA_{1-x}Gua_x)_2Pb_3Br_{10}$ and d) $BA_2(MA_{1-x}EA_x)_2Pb_3I_{10}$ 2D RP HMP films. This figure corresponds to a magnified view of the XRD data shown in Figure 1 of the main manuscript, focusing on the 13°–31° 2θ region to highlight the evolution of diffraction peaks with increasing A-site cation substitution. The indexing and phase assignments (n = 1, 2, 3) are discussed in the main text.

Table S1. PLQY (%) of the mixed A-cation $BA_2(MA_{0.40}A_{0.60})_2Pb_3I_{10}$ and $BA_2(MA_{0.40}A_{0.60})_2Pb_3Br_{10}$ 2D MHP thin films.

	I	Br
Ref	0.09 %	5.00 %
60% Gua	0.10 %	17.00 %
60% EA	0.04 %	0.50 %

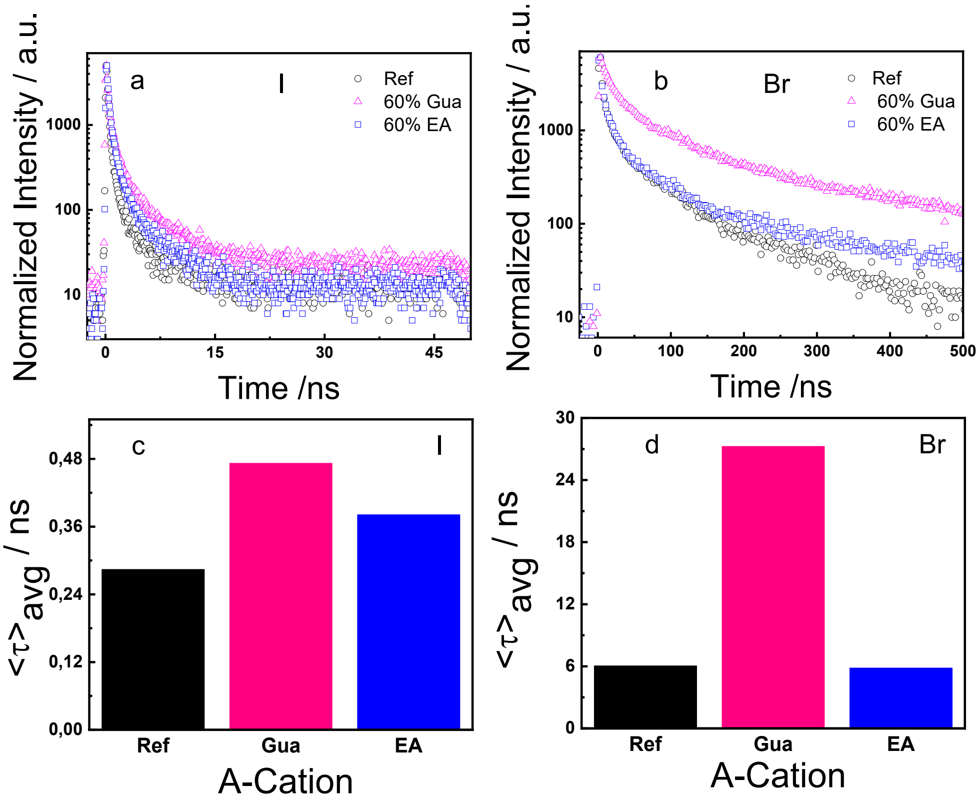

Figure S2. Panels a and b display the PL decays of $BA_2(MA_{0.40}A_{0.60})_2Pb_3I_{10}$ and $BA_2(MA_{0.40}A_{0.60})_2Pb_3Br_{10,}$ respectively, while panels c and d correspond to their average lifetime.

References

1. Ramos-Terrón, S., Jodlowski, A. D., Verdugo-Escamilla, C., Camacho, L. & De Miguel, G. Relaxing the Goldschmidt Tolerance Factor: Sizable Incorporation of the Guanidinium Cation into a Two-Dimensional Ruddlesden-Popper Perovskite. *Chem. Mater.* **32**, 4024–4037 (2020).
2. Ramos-Terrón, S., Verdugo-Escamilla, C., Camacho, L. & de Miguel, G. A-Site Cation Engineering in 2D Ruddlesden–Popper $(BA)_2(MA_{1-x}A_x)_2Pb_3I_{10}$ Perovskite Films. *Adv. Opt. Mater.* **9**, 1–15 (2021).
